# Supplementary material for: Are carbon emissions trading and green financial instruments synergistic? -Comprehensive quantitative research based on content analysis
Source: PLoS One. 2024 Mar 7;19(3):e0298601. doi: 10.1371/journal.pone.0298601 (PMC10919601; doi:10.1371/journal.pone.0298601)
Supplement: S1 Data — (ZIP) [file pone.0298601.s001.zip › Data/地级市Z_F工作报告.zip!/╜¡╦╒/╒≥╜¡╩╨/└┤╫╘╣1⁄2╓┌║┼í╛┬φ┐╦╩2╛▌═°í┐_2010.docx]

各位代表：

　　现在，我代表镇江市人民政府，向大会作工作报告，请予审议，并请市政协委员和其他列席人员提出意见。

　　去年工作回顾

　　刚刚过去的2009年，令人难忘。

　　一年来，在市委的正确领导下，我们坚持以科学发展观统领全局，认真贯彻中央和省决策部署，紧紧依靠全市人民，积极应对金融危机的深度影响，危中抢机、逆势而上、奋力拼搏，除进出口指标外，全面完成市六届人大二次会议确定的各项任务。预计全市实现地区生产总值1580亿元，同比增长13.5%；地方财政一般预算收入101.6亿元，增长18.6%；全社会固定资产投资1010亿元，增长40.6%；实际利用外资13.2亿美元，增长10%；社会消费品零售总额490亿元，增长19.5%；城镇居民人均可支配收入21240元，实际增长12%；农民人均纯收入9695元，实际增长12.1%；城镇登记失业率2.35%；单位GDP能耗下降4.65%，化学需氧量削减2.2%，二氧化硫排放量削减4%。全社会固定资产投资、实际利用外资等多项主要经济指标增幅一直位居全省前列。“抢抓机遇、跨越发展”成为过去一年镇江最鲜明的特征。

　　1．抢抓产业振兴机遇，结构调整取得较好进展

　　主动对接国家和省产业振兴规划，加快推进结构调整。“千百亿工程”深入推进。装备制造等五大产业实现销售2515亿元，增长14.8%，占工业总量的83.6%。10个项目列入全省100个重大产业项目计划，一大批薄膜太阳能、风能、核能及航空装备、新型节能环保项目成功落户。签定央企项目10个，总投资220亿元。金东、大亚、奇美3家企业销售超百亿元。“千企技改”全面实施，完成技改投入480亿元，占全部工业投入的80%。高新技术产业加快发展。实现产值1063亿元，占规模以上工业的比重达33.1%。建成科技孵化中心25万平方米，镇江大学科技园升格为国家级。全市科技进步贡献率达51%，第七次被评为“全国科技进步先进市”。“331人才计划”顺利实施，34个新兴产业项目、172名高层次人才得到资助。现代服务业加速提升。增加值占GDP的比重达37.7%。软件产业营业收入达65亿元，增长116.7%；成为省级国际服务外包示范城市；金山大剧院、沃尔玛超市等一批项目建成开业；商品房销售457.3万平方米，增长113.6%；私人轿车新上牌数突破3万辆，增长71.7%；旅游收入增长26%。现代农业提速增效。完成“三资”投入农业34.5亿元。新增高效农业22.4万亩，其中设施农业5.8万亩，高效农业面积占耕地面积的34.9%，农民现金收入中16.8%来自高效农业。4个中型水库除险加固项目争取中央扩大内需资金9034万元，丹阳、扬中通过省级农村河道疏浚验收。

　　2．抢抓扩大内需机遇，城乡面貌发生显著变化

　　利用国家实施4万亿投资的有利时机，一手抓拆迁，一手抓重大基础设施建设，投资量、拆迁量和建设工程量均创历史新高。完成城乡建设投资305.1亿元，同比增长31.7%。实行依法、阳光、惠民、和谐拆迁，市区完成拆迁381万平方米，增长1.1倍。南徐新城、北部滨水区、大市口AB地块、双井路片区和“三铁一桥”等建设顺利推进，金港大道、南山西入口、新南门汽车站、滨江旅游专线等工程顺利竣工，完成50万平方米老住宅小区整治。开通两条大运量公交线，完成出租车更新，城市照明控制中心建成启用。城管重心全面下移，“数字城管”正式运行。7个省级开发区基础设施投入98亿元，增长75.3%。在全省率先试点万顷良田建设工程。农村“三集中”有序推进，新建农民集中居住点63个，面积257万平方米。农村区域供水入户率、卫生户厕普及率、行政村班车通达率、互联网接入率进一步提高。家电下乡销售7.98万台，获财政补贴2038万元。生态市创建全面推进，“青山绿水”工程加快实施，21个乡镇通过全国环境优美乡镇省级考核，秸秆禁烧效果明显好于往年，已建和在建污水处理厂34座，太湖流域140项重点整治工程基本完成。

　　3．抢抓宏观政策机遇，改革创新力度不断加大

　　充分利用国家实施积极的财政政策和适度宽松的货币政策的宏观环境，筹措资金、深化改革、推进开放。获得中央预算内和省级配套专项资金5.17亿元，发行企业债券30亿元，BT融资40亿元。金融机构本外币各项贷款余额1348.3亿元，新增贷款382.6亿元，均创历史之最。华夏、民生、浦发、大新、招商等5家银行分支机构落户镇江。成立5家中小企业金融服务中心，“淡马锡”融资模式为540户中小企业发放贷款31亿元。成立8家农村小额贷款公司。新增7家创业投资公司。利华国际在纳斯达克成功上市。调整市区财税分配关系，顺利实施市直行政事业单位零基预算。市县政府机构改革、开发区综合改革、军转干部安置方式改革、市属事业单位分类改革和国企改革扫尾工作有序推进。农村改革强力推进，新增合作组织531家，新增入社农户4.2万户。涉农乡镇全部建立土地流转服务中心。

　　4．抢抓保障民生机遇，社会事业协调全面发展

　　认真落实中央和省一系列惠民政策，加快改善民生。脱贫攻坚“两消除”任务全面完成。镇江被评为首批“中国创业之城”，新增私营企业4828家、个体工商户1.82万户，累计注册资本达661亿元；新增城镇就业5.2万人，转移农村劳动力3.3万人，返乡农民工基本实现再就业；镇江籍高校毕业生就业率达90%。实现低收入住房困难家庭应保尽保。构建起“两基本一补充”社会养老保障体系，城乡医保人口覆盖率92.3%。人口出生缺陷发生率降至9‰。成为全省唯一公立医院改革试点城市，成立两大医疗集团，医药卫生体制改革取得良好开端。新二院整体搬迁工程、一院内科医技楼、中医院门诊综合楼建设加快推进。公共卫生服务能力显著增强，有效防控甲型H1N1流感疫情。全面完成县级区域教育现代化创建，率先发放义务教育绩效工资。文化产业发展迈开步伐，新广电中心、金山演艺广场、西津渡老码头文化创意园投入使用，成功举办“大爱镇江”、“万人红歌会”、“迷笛音乐节”和市民大讲堂等活动，数字电视整体转换率达92%。体育会展中心、职工文体活动中心等开工建设，竞技体育和全民健身运动蓬勃开展。“平安镇江”、“法治镇江”建设深入推进，再次获得“全国社会治安综合治理优秀地市”称号。信访工作扎实有力，为全局发展作出重要贡献。援建绵竹板桥镇的卫生院等5个重点工程交付使用。双拥工作再创佳绩，安全生产、食品药品安全和民族宗教、外事、侨务等工作取得新进展。

　　5．抢抓“效能建设”机遇，行政水平进一步提升

　　坚持创新思路。树立“产业兴则镇江兴”、“结构调整定输赢”的工作导向，全力推进结构调整。深入研究长三角一体化新格局下加快提升城市软硬实力的具体办法。坚持克难奋进。实施“保增长促发展”六大工作机制，开展“服务中小企业千户行”等活动，及时出台扶持房地产、外贸发展等政策，完善土地收储办法，加强投融资平台建设，有效解决了资金、用地等难题。坚持真抓实干。开展招商、推进项目、组织拆迁、建设工程等各项工作，都做到只争朝夕、责任到人、取信于民。坚持依法行政。建立权力阳光运行系统和大督查机制，在全省率先开通市政府“12345”服务热线，继续深化“两集中、两到位”工作，切实加强对重要领域、重点项目和资金使用的审计监督。自觉接受人大监督，认真执行人大决议决定，主动加强与政协的民主协商，办理人大代表建议219件、政协提案459件，满意率达97%以上。充分听取人大代表和政协委员的意见建议，做到科学民主决策，切实依法行政。

　　伴随着城市的发展与变化，我们欣喜地看到：在实干兴市的氛围中，在经济指标的升位中，在城乡面貌的巨变中，在发展成果的共享中，镇江人坚定了跨越发展的信心，激发了建设家园的热情，找到了苏南城市应有的自豪感，迎来了大刀阔斧干事业的新时代。现在的镇江，城市充满了活力，人民满怀着希望！

　　在此，我代表市人民政府，向全市人民，向所有关心支持镇江发展的社会各界，表示衷心的感谢并致以崇高的敬意！

　　在总结成绩的同时，我们也清醒地看到，经济社会发展中还存在不少薄弱环节。主要是：经济结构不优，新兴产业占比偏小；城市创新能力不强，综合实力偏弱；城乡居民收入水平偏低，对消费的拉动作用不强；城市管理需要进一步加强；等等。这些问题，我们将在今后的工作中，采取有力措施，认真加以解决。

　　今年主要工作

　　今年政府工作的总体要求是：认真贯彻党的十七届四中全会、中央经济工作会议以及市委五届九次全会精神，坚持“跨越发展、创新驱动、绿色增长、和谐共享”，以转变发展方式为主线，以全面加速城市化为手段，加快结构调整，完善城市功能，推进城乡一体，切实改善民生，促进社会和谐，建设充满活力、古代文明与现代文明交相辉映的现代化山水花园城市。

　　全市经济社会发展的主要预期目标是：地区生产总值增长12%；地方财政一般预算收入增长15%；全社会固定资产投资增长20%；社会消费品零售总额增长17%；实际利用外资增长10%；研发投入占GDP比重达到2%，高新技术产业产值占规模以上工业比重达到35%；城镇居民人均可支配收入、农民人均纯收入增长10%；城镇登记失业率控制在4%以内；单位GDP能耗、化学需氧量和二氧化硫排放量全面完成“十一五”削减任务。

　　围绕上述目标，我们将全力抓好以下六个方面：

　　（一）推进经济结构调整，增强城市实力

　　坚持转变方式、创新驱动，乘势而上、强抓投入，确保全年产业类投资完成850亿元。

　　大力发展新兴产业。围绕形成规模、产值增长40%以上的目标，在新能源领域重点发展薄膜太阳能和核电、风电装备制造，薄膜太阳能形成从设备制造、电池生产、TCO玻璃、发电站到农业发电大棚的产业链，核电、风电装备制造形成核心部件、整机制造及配套能力；在新材料领域重点进行碳纤维、高温合金等的研发生产，形成集聚优势；在电子信息领域重点发展物联网、智能电网和集成电路；在海洋工程领域重点发展船舶设计和核心部件制造以及深海钻井平台配套等。

　　推动支柱产业升级。通过引进技术、自主研发、改进工艺与装备等途径，加速产业升级，加快推进“千百亿工程”向纵深发展，五大产业实现销售增长20%以上，新增3家销售超百亿元企业。新增省级高新技术企业50家，省级高新技术产品120个，高新技术产业产值突破1400亿元。继续实施“千企技改”工程，完成技改投资600亿元，坚决淘汰落后产能。大力开展节能降耗、清洁生产和循环经济试点，构建秸秆生物质能开发利用等循环经济产业链，积极探索“绿色制造”和低碳经济发展路子。

　　加快发展现代服务业。力争服务业增加值占GDP比重达到40%。引进和新建薄膜太阳能、电力电器、智能电网等10家省级以上研发机构和检测中心，新建100万平方米科技孵化中心。加快西津渡文化产业园、南山文化休闲园等园区建设，吸纳各类资本推动文化产业大发展。着力推进大港四期工程和中石油长江项目、惠龙国际等物流基地建设，新建现代物流仓库20万平方米。深入实施“软件千企”工程，加快服务外包产业发展。完善市场政策，保持房地产业稳定健康发展。改造提升传统商业业态，新建高端商业场所100万平方米。按照把旅游业培育成为战略性支柱产业的要求，加大投入力度，主动对接上海世博会，开工建设8家左右五星级标准酒店，积极推动“三山”、茅山创建国家5A级旅游景区，开通一批乡村旅游专线。

　　积极发展现代农业。加快土地流转步伐，建设3—5个核心区域1万亩以上的现代农业园区，新建投资1000万元以上的高效农业规模基地50个，新增高效农业22万亩、其中设施农业5.5万亩。加强市场建设，强化科技支撑，突出为农服务，培育现代农民。坚持量质并举，大力发展“四有”合作经济组织，新增入社农户占比达25%以上。全面完成县乡主要河道疏浚和中小水库除险加固工程。

　　继续提升开放水平。切实加大招商选资力度，力争新批外资大项目15个以上，投资超亿元的央企项目10个以上、民资项目80个以上。创新利用外资方式，大力引进创业投资基金、股权基金。推动各开发区由规模化产业集群向高效化创新集群转型，由单一功能区向叠加功能区转型，由外延式扩张向可持续发展转型，推进镇江经济开发区升格为国家级。大力优化出口产品结构。继续实施创新创业领军人才集聚工程，引进10个以上领军团队、30名以上领军人才，引进硕士以上人才1000名。继续开展国际友好交流等活动，提升镇江知名度。

　　（二）树立现代城市理念，提升城市品质

　　围绕凸显山水花园城市的特色和个性，切实提升主城的规划、建设和管理水平。

　　构筑“一中心四区域”大格局。大市口核心圈进一步强化中央商务区功能，加快推进大市口A、B地块和双井路片区等建设，提升周边环境。南徐新城以办公、居住、康体功能为主，新行政中心、档案馆、市民公园、文化公园和新二院一期工程交付使用，商务办公A区、体育会展中心、职工文体中心和市场服务中心综合楼主体封顶，开工建设商务办公B区、交通综合楼和高铁场站，花园城基本成型。北部滨水区凸显文化、旅游功能，全面完成内江控水各项工程，贯通滨江外环线，实施内江北岸景观改造，启动文化艺术中心、长江文化主题公园和西津湾不夜城建设，着力打造长江最美的城市港湾和江南最美的城市名片。丁卯科技城致力于打造新兴产业功能区，新增科技创新载体50万平方米以上，建成科技园中心研发区、专家公寓酒店和1号研发楼，确保2号研发楼主体封顶，开工建设产业集聚A区等项目。南山——官塘板块强化生态、休闲、商住功能，整治南山周边环境，完成南山森林防火通道和旅游绿色通道建设，建成开放莲花洞景区，促使南山成为城中“绿肺”；完成官塘片区控制性规划编制，有序推进基础设施建设和土地一级开发整理。

　　推动城市品位快提升。进一步提升规划水平，坚决做到规划不成熟、不动一寸土。实施5个危旧房片区和10个城中村改造，完成50万平方米老小区成片整治，基本完成市区菜市场改造任务，推进邻里中心建设，在各类新建筑群和风光带同步配套公共服务设施。完成市区高速公路三大出入口整治，建设苏宁、万达、九润等一批融购物、休闲、商住等为一体的城市综合体，开工建设一批高层或超高层建筑，提升城市形象，丰富城市天际线。提高建筑形态、风格和色调的设计水平，实现长江路、南徐大道、中山路和解放路等重点地段形象设计全覆盖。进一步加快西津渡等历史文化街区的保护性开发，传承城市文脉。

　　推进精致管理全覆盖。积极应用信息技术、物联网技术，启动“智慧城市”建设。提高数字城管水平，完善城管执法体制，落实城管重心下移责任，增强实时监测与动态控制能力。整治违章搭建和占道经营，强化对各类工地和渣土运输的管理，防治二次扬尘污染。对南徐大道、金港大道、中山路、解放路和长江路等干道实施智能化管理，新增一批停车泊位，缓解交通拥堵和停车难问题。实施无物管小区集中整治，落实长效管理措施。

　　（三）推动城乡一体发展，扩展城市空间

　　加快城市组团发展。顺应当前城市之间集群化、互补性、联动式发展的趋势，强化主城区的辐射带动作用，促进丹阳向中等规模城市发展，句容与南京同城同建，扬中向全岛城市化迈进，丹徒新城与主城无缝对接，大港板块建设滨江产业生态新城。

　　优化城镇布局规划。加快城郊结合部镇改街道、村改社区的步伐，促进农民变市民，推进土地等资源集约利用。深化农村综合配套改革，以城乡挂钩、万顷良田工程和“双置换”、“三集中”为手段，建设10个左右新市镇、新社区示范点和30个左右中心村集中型新社区，推进未成年人社会实践基地建设。

　　统筹基础设施建设。加快主城区“三横九纵”路网建设，基本消除丁字路、断头路，打通交通节点，做到“外部高速成环、内部加密成网”。继续推进“三铁一桥一枢纽”等重大项目建设，开工建设泰州大桥东、西高速接线工程，实现全市域半小时内上高速目标。实施338省道、238省道新区段拓宽改造工程。加快推进公交全覆盖，建成城际综合客运枢纽站，开通大运量公交3、4号线，建成丹徒新城、镇江新区等公交停车场和枢纽站。推进农村公路及客运班车向较大自然村延伸。加快天然气管网向乡镇延伸，完成区域供水管网263公里，解决农村10万人饮水安全问题。

　　（四）加强生态文明建设，彰显城市特色

　　全力推进生态市创建。坚持整治与建设并重，夯实生态市建设基础，达到国家生态市创建标准。句容、丹徒、丹阳和扬中达到国家生态县建设标准，全市80%以上的乡镇达到环境优美乡镇标准。加强环保基础设施建设，34座污水处理厂形成减排能力，城市污水集中处理率达到85%以上，47个村污水处理设施完成建设任务。加大饮用水源地保护力度，治理农业面源污染，确保集中式饮用水源水质达标率为100%。突出抓好重点行业和企业的环境整治，加强机动车尾气污染防治。

　　全面完成“青山绿水”工程。全部拆除主城区山体周边和河道两侧的违法建设和不符合规划的破旧建筑，退房还山、修复生态、完善景观，完成11座山体和古运河中段等3条通江河道整治，新增12个开放式公园，实现山水城林融为一体。

　　全民行动美化家园。在主城区重点抓好垂直绿化、精细绿化和小区绿化，大力建设城区小游园，努力做到相距500米有绿地；在辖市大力推进村庄以及沿铁路、公路和河道的绿化，确保全年植树造林10万亩。深入推进农村环境卫生长效管理，进一步提升镇村环境面貌。

　　（五）提高人民生活品质，促进城市和谐

　　建设创业城市。培育创业文化，大力推动自主创业、全民创业。加强创业载体建设，每个辖市建设1个高标准综合性创业孵化基地，高校建设一批创业基地。建成苏南人力资源市场，建立四级创业指导服务体系。全年成功扶持创业6500人，带动就业3.5万人，争创国家级“创业型城市”。坚持创业带动就业，突出抓好大中专毕业生、城镇就业困难群体、农村劳动力和被征地农民就业。完善零就业家庭动态清零长效机制，率先建成“充分就业市”。

　　建设幸福城市。同步推进社保制度和参保人群两个“全覆盖”，实施“五险合一”统一征缴，企业职工基本养老保险新增扩面3万人，职工参保率达95%以上，城乡居民养老保险覆盖率达70%以上。有序将新农合整体纳入城乡居民医保制度，实现城乡居民医保一体化运行，全市各类医疗保险覆盖率达95%以上。加强廉租房、经济适用房和公共租赁房等保障性住房建设。全面完成以公立医院改革为核心的医药卫生体制改革，认真落实国家基本药物零差率制度，继续减轻群众就医负担。加强对甲型流感等各类传染病的防控，完善突发公共卫生事件应急机制。继续加大对社会困难群体的救助力度，大力发展老龄和慈善事业。继续做好扶贫和茅山老区开发工作。

　　建设人文城市。以争创全国文明城市为抓手，提升市民文明素养，增强城市软实力。积极实施“文化营销城市”策略，提升“文化嘉年华”品牌，开展“文心公益行动”，举办“长江迷笛音乐节”，推动大运河申遗，催生新兴文化业态，加大城市形象宣传力度。切实推进义务教育均衡发展，加大中小学校舍安全工程推进力度，加快教育资源整合和布局调整，推进更高水平的教育现代化。广泛开展全民健身活动，积极备战省第十七届运动会。加大“双拥”六创全国模范城力度，扎实推进“双拥”八大工程，切实做好外事、侨务、人口计生、民族宗教和妇女儿童等工作。

　　建设平安城市。密切关注企业的生产经营，依法保护职工权益，防止经营风险转化为社会风险。严格落实安全生产责任制，减少一般事故，遏制较大事故，杜绝重特大事故，实现事故起数、死亡人数连续九年“双下降”。大力推进质量兴市战略，强化食品药品监管，规范市场经济秩序。加强社会治安综合治理，做好“五五”普法末期考核验收工作，积极创新社会管理机制，切实提升技防水平和应急能力，依法严厉打击各种刑事犯罪和经济犯罪，认真做好信访和矛盾调解工作，扎实推进“法治镇江”、“平安镇江”建设。

　　（六）加强政府自身建设，引领城市升级

　　城市管理者的眼光，决定着城市的未来。加快城市化，必须进一步提升政府工作水平。

　　坚持开放创新。全体公务人员将按照建设学习型政府的要求，加强学习，努力以全球观念和战略眼光，研究城市化和城乡一体化的规律，更加热爱城市、熟悉城市，更好地建设城市、管理城市。自觉树立市场观念和开放意识，善于依据市场规律经营城市；完成新一轮政府机构改革，继续深化投融资体制改革，全面放开城市建设各类市场，引进更多的外来投资者参与城市化进程。

　　坚持以人为本。在加速城市化进程中，一定更加注重利民、便民、亲民。联动推进户籍等配套制度改革，让城市化为人民群众提供更多的安身之所、生活之便、创业之需和富裕之路。继续坚持依法、阳光、惠民、和谐拆迁，确保人民群众的合法权益；始终把人民群众的需求放在第一位，每项工作的安排与推进，每个工程的设计与建设，都自觉做到以满足人民、服务人民为根本。

　　坚持依法行政。市政府及工作部门将始终坚持依法行政、公正透明。认真执行市人大及其常委会的决议和决定，定期报告工作，自觉接受监督。不断加强与市政协的民主协商，主动听取各民主党派、工商联、无党派和社会各界人士的意见。对事关群众利益的重大决策，深入研究论证，广泛听取意见。

　　坚持廉洁从政。严格落实党风廉政建设责任制，深入推进行政权力网上公开透明运行，严肃查处违法违纪行为。强化审计监督和行政监察，带头厉行节约，保持政府机关清廉、为民、务实的良好形象。

　　各位代表，2010年是充满挑战而又令人期待的一年。让我们在市委的坚强领导下，团结和依靠全市人民，继续开拓创新、真抓实干、合力攻坚，为全面加速城市化、提升竞争力，为实现镇江跨越发展、后发先至而努力奋斗！
